# Low-Temperature Synthesis of Anatase TiO_2_ Nanoparticles with Tunable Surface Charges for Enhancing Photocatalytic Activity

**DOI:** 10.1371/journal.pone.0114638

**Published:** 2014-12-15

**Authors:** Ye Li, Zhenping Qin, Hongxia Guo, Hanxiao Yang, Guojun Zhang, Shulan Ji, Tingying Zeng

**Affiliations:** 1 College of Environmental and Energy Engineering, Beijing University of Technology, Beijing, China; 2 College of Materials Science and Engineering, Beijing University of Technology, Beijing, China; 3 Beijing Key Laboratory for Green Catalysis and Separation, Beijing, PR China; 4 MIT, Elect. Res. Lab, Cambridge, Massachusetts, United States of America; RMIT University, Australia

## Abstract

In this work, the positively or negatively charged anatase TiO_2_ nanoparticles were synthesized via a low temperature precipitation-peptization process (LTPPP) in the presence of poly(ethyleneimine) (PEI) and poly(sodium4- styrenesulfonate) (PSS). X-ray diffraction (XRD) pattern and high-resolution transmission electron microscope (HRTEM) confirmed the anatase crystalline phase. The charges of the prepared TiO_2_, PEI-TiO_2_ and PSS-TiO_2_ nanoparticles were investigated by zeta potentials. The results showed that the zeta potentials of PEI-TiO_2_ nanoparticles can be tuned from +39.47 mV to +95.46 mV, and that of PSS-TiO_2_ nanoparticles can be adjusted from −56.63 mV to −119.32 mV. In comparison with TiO_2_, PSS-TiO_2_ exhibited dramatic adsorption and degradation of dye molecules, while the PEI modified TiO_2_ nanoparticles showed lower photocatalytic activity. The photocatalytic performances of these charged nanoparticles were elucidated by the results of UV-vis diffuse reflectance spectra (DRS) and the photoluminescence (PL) spectra, which indicated that the PSS-TiO_2_ nanoparticles showed a lower recombination rate of electron-hole pairs than TiO_2_ and PEI-TiO_2_.

## Introduction

Photocatalytic degradation is an efficient and economical method to totally decompose organic contaminants into benign substances [1]–[2]. Titanium dioxide (TiO_2_) nanocrystal has attracted much attention as a photocatalyst due to its photostability, nontoxicity, high activity and relatively low cost [3]. TiO_2_ has three crystalline forms: rutile, anatase and brookite [4]. Anatase titania usually exhibits higher photocatalytic activity than rutile and brookite, owing to its higher density of localized states, the surface-adsorbed hydroxyl radicals and slower charge carrier recombination [5]–[7]. Much work has been reported for preparation of anatase TiO_2_, such as thermolysis [8]–[9], sol-gel [10]–[11], ultrasonic technique [12], solvothermal [13] and hydrothermal method [14]–[15], by which the resulted particles have to be annealed at suitable temperature to obtain crystalline TiO_2_ with anatase phases. The annealing process at relatively high temperature would inevitably lead to particles agglomeration and hence reduction of their specific surface area [16]–[17]. Therefore, the low temperature methods for preparation of anatase TiO_2_ particles and films attract much attention, due to the fact that calcinations step can be eliminated. Mesoporous aggregates of anatase TiO_2_ were obtained by controlled hydrolysis reaction of titanium tetrabutoxide in non-aqueous media, isopropanol, and then aging at ambient conditions for more than 120 days [18]. Gao et al. have synthesized the superfine TiO_2_ nanocrystals with tunable anatase/rutile ratios in aqueous ethanol solution by “low temperature dissolution–reprecipitation process” (LTDRP) and solvethermal treatment [19]. Burunkaya et al. have prepared directly the anatase TiO_2_ particles by hydrolyzing titanium ethoxide in alcohols solvent, such as ethanol, n-butanol and n-hexanol, followed by reflux at low temperature of 93°C, without calcination step [20]. An anatase hydrosol has been obtained by acidic hydrolysis of titanium isopropoxide at room temperature [21]–[22], and the nanocrystalline TiO_2_ films were prepared after adding surfactant into the hydrosol. However, all of these anatase TiO_2_ particles or films have been prepared using titanium alkoxide or chloride in non-aqueous alcohol media.

Moreover, the TiO_2_ suspension with a high degree of dispersion stability is considered one of the important factors during the photocatalytic processes [23]–[27]. Thus, modification of TiO_2_ particles by polymers or surfactants, which combines unique properties of dissimilar components, would favor dispersing the suspension. Two ways have been used to modify TiO_2_ particles: one is to introduce a surfactant or polymer to a preformed nanoparticle (post-synthesis) [23]–[24]; and the other is to incorporate surfactants or polymers simultaneously during nanoparticle synthesis (in situ synthesis) [25]–[27]. The anionic surfactant, such as sodium dodecylsulphate (SDS) and sodium dodecyl benzene sulfonate (SDBS), have been introduced into TiO_2_ particles for promoting the dimerization of rhodamine B (RhB) and subsequent adsorption on the TiO_2_ surface [26]–[29]. The nanosized titanium dioxide particles and charged polyelectrolytes (PEs), such as poly(allyl aminehydrochloride) (PAH) and poly(styrene sulfonate sodium) (PSS), have been alternatively assembled to form multilayer composite film for photodegradation of RhB under ultraviolet (UV) irradiation [30]. However, the charge controllability and the photocatalytic performance of the positive or negative TiO_2_ particles modified by polyelectrolyte need further investigation.

In this work, the charge-tuned TiO_2_ nanoparticles with pure anatase phase were synthesized via a low temperature precipitation-peptization process (LTPPP) under ambient condition. The water soluble titanium sulfate salt (Ti(SO_4_)_2_) was used as precursor to fabricate either positive or negative anatase TiO_2_ nanoparticles in the presence of cationic or anionic polyelectrolytes (PEs). The charges of as-prepared nanoparticles, such as pure TiO_2_, poly(ethyleneimine) (PEI)-TiO_2_ or poly(sodium 4-styrenesulfonate) (PSS)-TiO_2_, manifested by zeta potentials, were tuned by the variations of pH and the amount of the polyelectrolytes, respectively. Then, the photocatalytic activities of as-prepared TiO_2_ nanoparticles were investigated by degradation of organic methylene blue (MB) and rhodamine B (RhB) dyes. The possible mechanism of the photocatalytic performance of the charged TiO_2_ nanoparticles is proposed based on photoluminscence (PL) emission and UV-vis diffuse reflectance spectra (DRS). This work may provide an additive strategy to charge controllable TiO_2_ nanoparticles, which would find new application in other fields, such as electrophoretic particles and the charged films or membrane [31]–[33], in addition to photocatalysis.

## Experiments

### 2.1 Materials

Titanium sulfate (Ti(SO_4_)_2_), methenamine(C_6_H_12_N_4_), nitric acid, ethanol (analytically pure), methylene blue (MB) and rhodamine B (RhB) dyes were provided by Beijing Chemical Factory (Beijing, China) and used without further purification. Poly(ethyleneimine) (PEI, Mw = 750,000) and poly(sodium4-styrenesulfonate) (PSS, Mw = 70,000) were purchased from Sigma- Aldrich Co.,(St. Louis, MO, USA). Deionized water was used throughout this research.

### 2.2 Preparation of PEs-modified TiO_2_ nanoparticles

The PEs-modified TiO_2_ nanoparticles were obtained at low temperature precipitation-peptization process (LTPPP). The negatively charged TiO_2_ nanoparticles modified by PSS were prepared as an example. Specifically, 2.4 g titanium sulfate, used as titanium precursor, was dissolved in 50 ml of deionized water; then PSS was added (titanium sulfate: PSS = 1∶0.15∼1∶0.05 mole ratio) under stirring at ambient condition until it thoroughly dissolved. Subsequently, the mixture containing titanium precursor and PSS was added into 50 mL of 2.7 wt% C_6_H_12_N_4_ aqueous solution in dropwise of 0.5 mL/min under stirring, and meanwhile, the precipitation produced. After stirring for 1 h, the precipitates were collected and washed with ethanol and deionized water thoroughly. Then the precipitates slurry was dispersed into 0.3 mol/L HNO_3_ aqueous solution, and refluxed for 3 h at 50°C. Thus, the PSS modified TiO_2_ (PSS-TiO_2_) was obtained, after thoroughly washed by water and dried. The process for preparing PEI modified TiO_2_ (PEI-TiO_2_) was similar as that of PSS-TiO_2_, using PEI instead of PSS polyelectrolyte. As a comparison, pure TiO_2_ nanoparticles were prepared in the same procedure without adding any additives.

### 2.3 Characterization

The crystallographic structures of the as-prepared nanoparticles were characterized by X-ray diffraction method on a D8 advance installation, (Bruker/AXS, Germany) using Cu−Kα1 (λ = 1.5406 Å) radiation source. Scanning election microscopy (SEM) (SU8020, Hitachi, Japan) was employed to observe the morphologies of the samples, and the EDX spectra of the corresponding region were obtained by the equipped Oxford EDX instruments and AZtec software. The microstructures of nanoparticles were explored by a high-resolution transmission electron microscope (HRTEM; JEM-2100, JEOL, Japan) equipped with an electron diffraction, operated at 200 KeV with LaB6 emission source of electrons. Zeta potentials and dimensions of the TiO_2_ nanoparticles in aqueous water suspension were measured using Zeta Potential/Particle Sizer (NICOMP^TM^ 380ZLS, USA), after ultrasonic for 60 min. The pH of the suspensions was adjusted using diluted HCl or NaOH solution.

The UV-vis diffuse reflectance spectra (DRS) were performed at room temperature on a UV-2450 instrument (SHIMADZU, Japan) in the range of 200 nm to 600 nm, with BaSO_4_ as the standard reflectance sample. The characteristic adsorption of the particles was obtained by the intercept of the linear extrapolation to the *λ* axis and the band gap energy (*E*_g_), given as a function of band edge (*λ*_g_) as formula (1), according to reference [34]–[35].



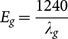
(1)


The photoluminescence (PL) spectra excited at 320 nm were recorded by a RF-5301PC fluorescence spectrophotometer (SHIMADZU, Japan) under ambient condition.

### 2.4 The adsorption performance and photocatalytic activity of the nanoparticles

The adsorption performances of the as-prepared nanoparticles were investigated by UV-vis intensity of 10 mg/L of methylene blue (MB) solution [36]. Specifically, 100 mg of photocatalyst, pure TiO_2_, PEI-TiO_2_, or PSS-TiO_2_, was added into 100 mL of Methylene blue (MB) solution of 10 mg/L of concentration, respectively. The resultant slurries were fully stirred in darkness for a given time. Then 5 mL of the suspension was taken out and centrifuged to remove the suspended solids within time intervals. The supernatant was subjected into the colorimetric ware for UV-vis analysis on a UV-vis spectrophotometer (UV-3200 MAPADA, Shanghai Mapada Instruments Co., Ltd., China).

The photocatalytic activity of the nanoparticles were evaluated by their photo degradation of 10 mg/L of methylene blue (MB) or rhodamine B (RhB) solution. A 500 W UV lamp (365 nm) was used as the light source, and its distance from the liquid level was 15 cm. The photo degradation on dyes begin after their sufficient adsorption on photocatalyst in darkness. In a typical procedure, 100 mg of photocatalytic particles was added into 100 mL of MB or RhB solution with 10 mg/L concentration, respectively. The resultant slurry was fully stirred in darkness until it reached adsorption/desorption equilibrium. Then, the suspension was placed under the irradiation of UV light. Within a given time, 5 mL of suspension were taken out and centrifuged to remove the suspended particles, and the supernatant was transferred into a colorimetric ware for UV-vis analysis on a UV-vis spectrophotometer (UV-3200 MAPADA, Shanghai Mapada Instruments Co., Ltd., China). The dye concentration was determined at a wavelength of 664 nm (for MB) and 554 nm (for RhB) and quantified by a standard calibration based on Beer-lambert's law. The photocatalytic activity of the samples was calculated according to *η* = ((*C_0_−C_t_*)/*C_0_*)×100%, where *η* represents the degradation efficiency of dyes, *C_0_* (mg/L) and *C_t_* (mg/L) represents the concentration of dye in the aqueous solution at time *t* = 0 and *t* respectively.

## Results and Discussions

### 3.1 Structure analysis on TiO_2_ nanoparticles

**Figure 1:**
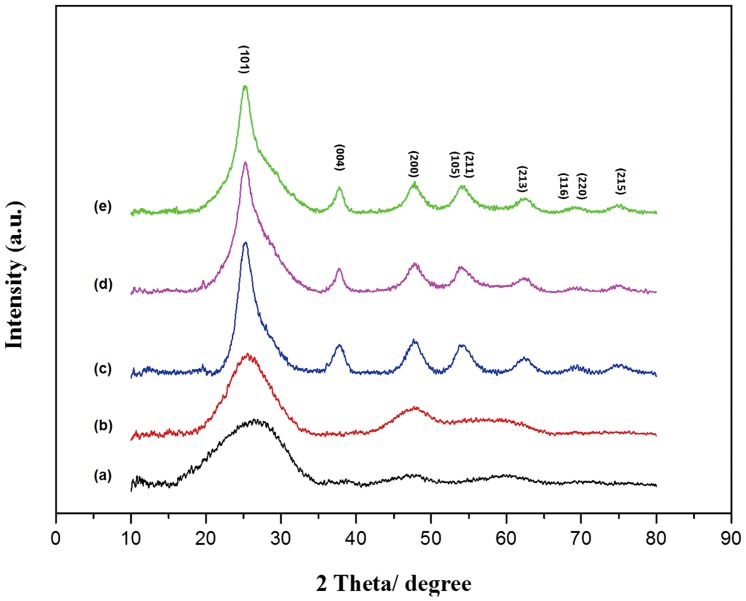
XRD patterns of pure TiO_2_ peptized at (a) 30°C, (b) 40°C and (c) 50°C; as well as the modified TiO_2_ of (d) PSS-TiO_2_ and (e) PEI-TiO_2_.

The anatase TiO_2_ nanoparticles were prepared by LTPPP method, including precipitation and peptization steps. First, the amorphous Ti(OH)_4_ precipitation was obtained by hydrolysis of titanium sulfate in the presence of alkaline methenamine. Then, the Ti(OH)_4_ precipitation was peptized by dehydration in HNO_3_ solution media, due to that the strong acidic environment favored formation of the single-crystalline anatase crystal [37]. The crystalline phases of pure TiO_2_ nanoparticles without any additives, peptized at temperature of 30°C, 40°C, 50°C, respectively, were first investigated by XRD patterns (Fig. 1). The XRD pattern of Fig. 1(a) showed an obvious wide peak at 2θ of 15.3–35.6° and the weak wide peak at 2θ of 42.4–52.3°, indicating the formation of amorphous crystal at relatively low temperature of 30°C. These two wide peaks become slightly narrowed in Fig. 1(b), indicating the formation of some primary crystallite grain, when the peptization was performed at 40°C. Then, the separate and sharp peaks at 2θ of 25.24°,37.7°, 48.1°, 53.8°, 55.0°, 62.6°, 68.7°, 70.2° and 75.0° were observed in Fig. 1(c), corresponding to (101), (004), (200), (105), (211), (213), (116), (220) and (215) planes of anatase TiO_2_ (JCPDS card No. 21-1272) [38], respectively. This indicated that the crystalline anatase nanoparticles were formed at temperature of 50°C. The size of pure TiO_2_ crystalline was calculated about 4∼6 nm by Scherrer equation [39]. The XRD patterns of the PEs modified nanoparticles at 50°C shown in Fig. 1(d)) and Fig. 1(e) indicated the similar peaks at 2θ position as that of the pure anatase TiO_2_. The average sizes of the PEI-TiO_2_ and PSS-TiO_2_ crystallines were estimated as about 9.0∼11.5 nm according to Scherrer equation.

**Figure 2:**
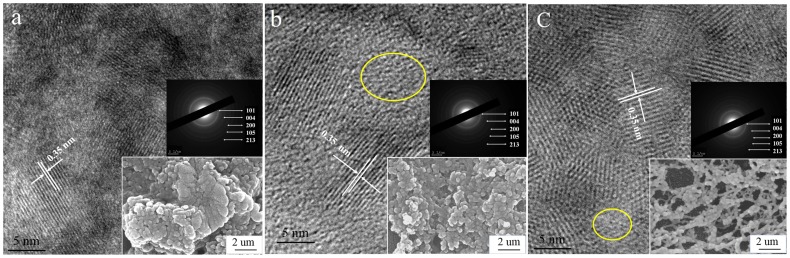
High-resolution transmission electron microscope (HRTEM) images, SEM images, and the selected-area electron diffraction (SAED) patterns of (a) pure TiO_2_, (b) PEI-TiO_2_ and (c) PSS-TiO_2_ nanoparticles.

Fig. 2 showed the high-resolution transmission electron microscope (HRTEM) images of pure TiO_2_, PEI-TiO_2_ and PSS-TiO_2_ nanoparticles. The enlarged TEM images in Fig. 2 showed that all three nanocrystallites have lattice fringes of 0.356 nm, corresponding to (101) faces of anatase crystalline, indicating that the introduction of PEI and PSS polyelectrolytes into the precursor had little effect on lattice structure of TiO_2_ crystal. Moreover, there were some amorphous regions in HRTEM images of Fig. 2(b) and Fig. 2(c), indicating that some immature crystalline in the presence of PEs. And the *d* spacing calculated from the electron diffraction pattern in the inset of Fig. 2 matched the values of the three as-synthesized TiO_2_ nanoparticles, which is in accordance with the consequence of XRD patterns of Fig. 1.

The SEM morphologies of the particles shown in the inset on the right bottom of Fig. 2 further manifested the overall morphology of nanoparticles. The SEM image of in Fig. 2(a) indicated that the pure TiO_2_ nanoparticles clumped and aggregated together, with diameter ranged from ∼200 to ∼500 nm. In contrast, the PEs modified nanoparticles in Fig. 2(b) and Fig. 2(c) were relatively uniform spherical with diameter about ∼150 nm to ∼200 nm; interestingly, the PSS-TiO_2_ nanoparticles were formed into some spherical bunched network. This indicated that the polyelectrolyte can reduce aggregation of the TiO_2_ nanoparticle, effectively.

**Figure 3:**
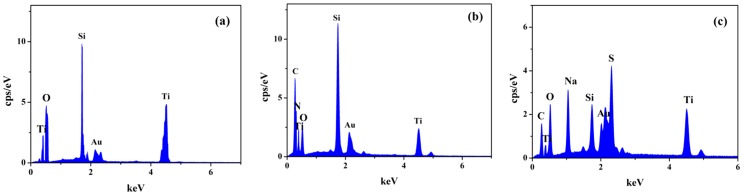
EDX spectra of (a) TiO_2_, (b) PEI-TiO_2_ (10%) and (c) PSS-TiO_2_ (10%).

EDX measurements further demonstrated the PEs modification on TiO_2_ particles. The existent elements on TiO_2_, PEI-TiO_2_ and PSS-TiO_2_ nanoparticles were shown in the spectra of Fig. 3, and the amounts of the elements were collected as in Table 1. Besides of peaks of the sprayed Au (2.1 keV) and Si (1.7 keV) of the silicon substrate, Fig. 3(a) indicated two peaks of Ti elements (0.4 keV and 4.5 keV) and O elements (0.5 keV), indicating the characteristic constituent of TiO_2_. In contrast, the new peaks of C (0.2 keV) and N (0.3 keV) elements in Fig. 3(b), and the peaks of C (0.2 keV), S (2.3 keV) and Na (1.0 keV) in Fig. 3(c) indicated obviously the existence of PEI and PSS on TiO_2_ particles. The amounts of these elements shown in Table 1 indicated that 29.22% of Ti and 70.78% of O were found on the pure TiO_2_ nanoparticles; while there were 33.73%, 66.27%, 34.62% and 13.18% of Ti, O, C and N elements, respectively, existing on PEI-TiO_2_ nanoparticle. Simultaneously, on PSS-TiO_2_ nanoparticle, the amount of Ti, O, C, S and Na elements were 22.86%, 28.11%, 26.15%, 12.15% and 10.73%, respectively. This clearly indicated that the modification and existence of PEI or PSS on TiO_2_ nanoparticles.

**Table 1:** The elemental amounts of TiO_2_, PEI-TiO_2_ (10%) and PSS-TiO_2_ (10%) nanoparticles.

nanoparticles	Elements content (wt%)					
	Ti	O	C	N	S	Na
TiO_2_	29.22	70.78	0	0	0	0
PEI-TiO_2_	33.73	66.27	34.62	13.18	0	0
PSS-TiO_2_	22.86	28.11	26.15	0	12.15	10.73

### 3.2 Zeta potential, particle size and dispersion stability of the nanoparticles suspension

**Figure 4:**
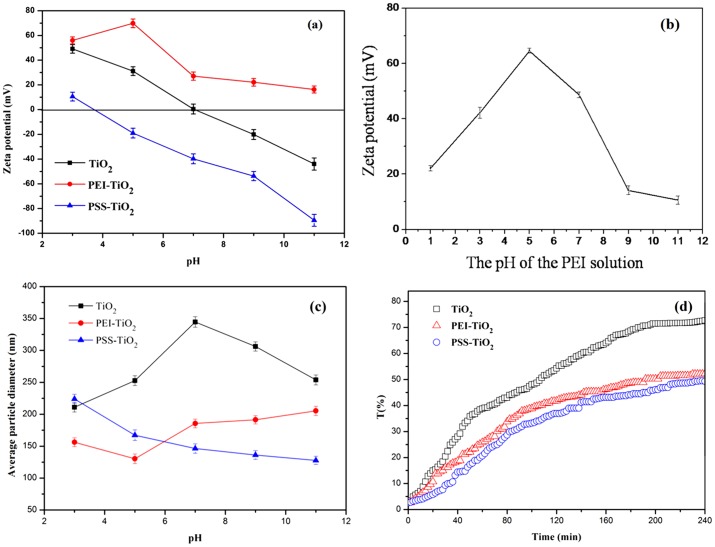
Zeta potential of pure TiO_2_, PEI-TiO_2_ (10%) and PSS-TiO_2_ (10%) as a function of pH (a); zeta potential of PEI (1.0 mg/mL) solution as a function of pH (b); Particle size of the TiO_2_, PEI-TiO_2_ and PSS-TiO_2_ nanoparticles at different pH (c); Transmittance changes of TiO_2_ (pH 3), PEI-TiO_2_ (pH 5) and PSS-TiO_2_ (pH 11) suspensions over time (d).

Fig. 4(a) showed the variation of zeta potential of the nanoparticles with pH increasing from 3 to 11. The zeta potential of the PEI-TiO_2_ nanparticles showed gradual decrease, except for the slight increase of PEI-TiO_2_ at pH of 5. The zeta potential of pure TiO_2_ nanopariticles varied from +49.16 mV to −44 mV with iso-electric point at pH of 7, similar as that of pure anatase TiO_2_ in Song' s work [40]. And zeta potential of the PSS-TiO_2_ showed minus increase from −16 mV to −89.48 mV. This was partly ascribed to the enhanced adsorption of hydroxyl ions on surfaces of the nanoparticles, yielding significant increase on the negative charge, similar as the change of zeta potential with the adsorption of Cl^−^, NO_3_^−^ and SO_4_^2−^ [41]. Moreover, the minus increase of zeta potential of TiO_2_ nanoparticles with pH is attributed to the process shown in formula 2, the change of TiOH_2_^+^ into TiO^−^ with pH increasing [28]:
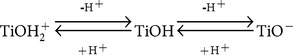
(2)For the PEI-modified TiO_2_ nanoparticles, the zeta potential showed a highest value of 69.85 mV at pH of 5.0. This was in accordance with the charge behavior of PEI, a weak polyelectrolyte, of which the amine groups were protonated in different level with pH [42], making it varied charges. As shown in the result of Fig. 4(b), the pure PEI showed a highest zeta potential at pH of 5, due to that its amine groups can be protonated enough at this pH value, resulting in the highest zeta potential of PEI-TiO_2_. The zeta potential of PSS, a strong polyelectrolyte, was little affected by pH of the solution [43]. Thus, the zeta potential of PSS-TiO_2_ is mainly dependent on the adsorptive abilities of hydroxyl ions on surfaces of nanoparticles with increase of pH, as well as the process shown in formula (2) [28], resulting in the highest zeta potential of −89.48 mV at pH of 11. Moreover, the variation of pH can also result in the change of the average dimension of the TiO_2_, PEI-TiO_2_ and PSS-TiO_2_ nanoparticles. As shown in Fig. 4(c), the TiO_2_ nanoparticles displayed largest average size at pH of 7, at which the zeta potential of the particle was zero (Fig. 4(a)). Thus, the almost no charged surface of the particles resulted in the aggregation more easily and with the largest aggregate size. In contrast, the PEI-TiO_2_ particle displayed smallest size at pH of 5. This was probably due to that PEI-TiO_2_ particle showed a highest zeta potential at pH of 5 (Fig. 4(a)), and the relatively strong electrostatic repulsion made the particles less aggregate and with smallest particle size. When pH value was greater than 5, the decreased zeta potential of PEI-TiO_2_ (Fig. 4(a) increased the chance of electrostatic attraction, and made the particles aggregate more easily and show the increased size. Similarly, the increased minus zeta potential of PSS-TiO_2_ particles with pH resulted in the gradually decreased size. This indicated that the variation of the average size of the particles with pH was much in accordance with that of zeta potential.

The dispersion stability of TiO_2_, PEI-TiO_2_ and PSS-TiO_2_ suspension were investigated by the variation of the suspension transmittance with time. The pH values of the three suspensions were 3, 5 and 11, respectively, in order to get a relatively higher charges and smaller aggregate size nanoparticles. As shown in Fig. 4(d), the transmittance of PEI-TiO_2_ and PSS-TiO_2_ suspensions displayed slower increase with time than that of pure TiO_2_ suspension, indicating that the relatively higher charges of nanoparticles can help to prompt the dispersion stability of the nanoparticles suspension.

**Figure 5:**
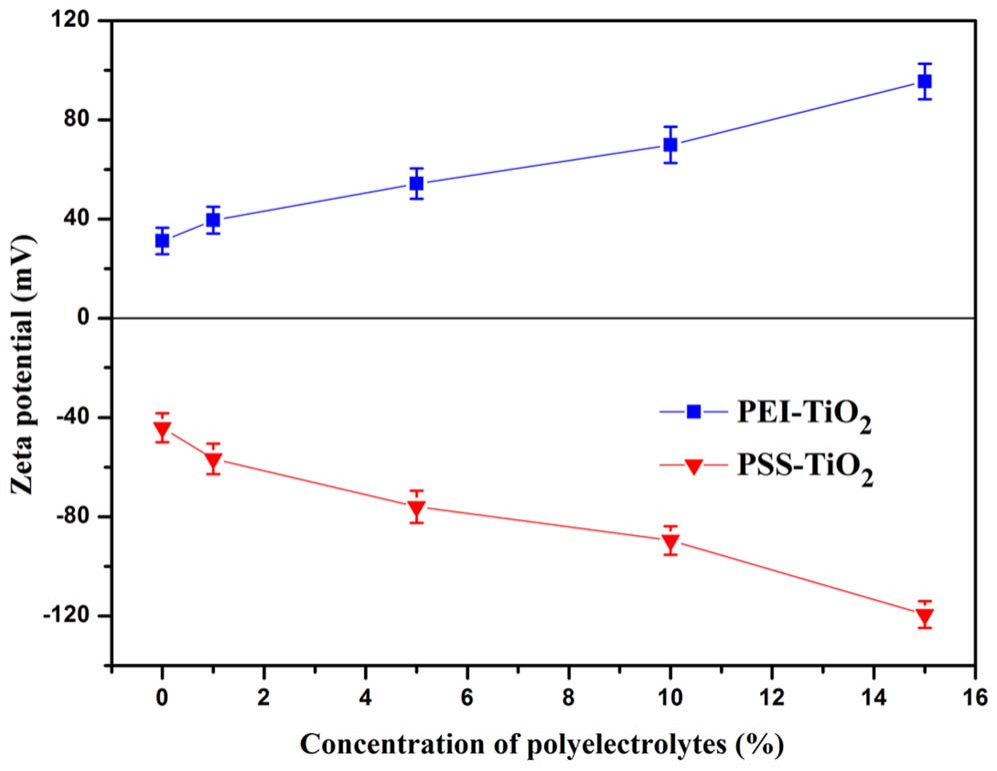
The variations of zeta potentials of PEI-TiO_2_ and PSS-TiO_2_ nanoparticles with concentration of polyelectrolytes (pH of PEI-TiO_2_ and PSS-TiO_2_ was 5 and 11, respectively).

In order to further achieve the tunable charge of nanoparticles, the variations of zeta potential with PEs concentration were investigated at pH value of the highest zeta potential of Fig. 4 (a). The results in Fig. 5 showed that the zeta potential of as-prepared PEI-TiO_2_ nanoparticles at pH of 5 significantly increased from +39.47 mV to +95.46 mV with concentration of PEI increased from 1.0 wt% to 15.0 wt%. Meanwhile, the zeta potential of the PSS modified TiO_2_ at pH of 11 showed obviously negative increase from −56.63 mV to −119.32 mV, with PSS concentration increased from 1.0 wt% to 15.0 wt%. The lower zeta potential of TiO_2_ in the PSS solution is in accordance with that in SDS solution [27]. This can be explained by the hemimicelle model [44]. At high pH of 11, the negative PSS was adsorbed on the TiO_2_ nanoparticle surface through physical adhesive interaction, causing the hemimicelle. When, PSS concentration increased, PSS micelles would be formed and adsorb on the TiO_2_ nanoparticle surface, and the increased adsorption volumes resulted in more negative zeta potential [28]. The similar elucidation can also be illustrated for the change of zeta potential of PEI-TiO_2_ with PEI concentration. The increases of zeta potential with the concentration of PEs indicated that the modification of polyelectrolyte of PEI or PSS on TiO_2_ nanoparticles resulted in the tunable surface charge of the particles, effectively.

### 3.3 Adsorption and photocatalytic activity of nanoparticles

#### 3.3.1 Adsorption of MB on the surface of nanoparticles

**Figure 6:**
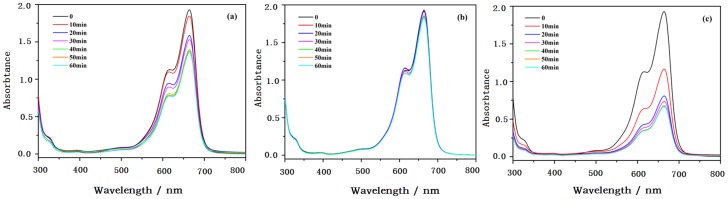
UV-vis spectra of MB by the adsorption on (a)TiO_2_, (b) PEI-TiO_2_ and (c) PSS-TiO_2_.

The adsorption of the organic substance on photocatalytic nanoparticles is considered as an important factor in their photocatalytic degradation. The adsorption performances of methylene blue (MB) on pure TiO_2_, PEI-TiO_2_, PSS-TiO_2_ were investigated by the absorbance changes of UV-vis spectra of MB at given time in darkness. The slightly decreased absorbance of UV-vis spectra in Fig. 6 (a) indicated that there was weak adsorption of MB on TiO_2_, which had little charges at pH of 7. And the absorbance the PEI-TiO_2_ suspension in Fig. 6 (b) showed almost no change, indicating the negligible adsorption of MB on positively charged TiO_2_. This was probably due to that the electrostatic repulsion between the positively charged PEI-TiO_2_ nanoparticles and the positively charged MB. In contrast, the absorbance of MB in Fig. 6 (c) showed obvious decrease indicating that there was more MB adsorbed on negatively charged PSS-TiO_2_ nanoparticles. This was due to that the positively charged MB was prone to adsorb on the negatively charged PSS-TiO_2_ through electrostatic attraction, which may favor the next photocatalytic process.

#### 3.3.2 Photocatalytic degradation of MB and RhB

**Figure 7:**
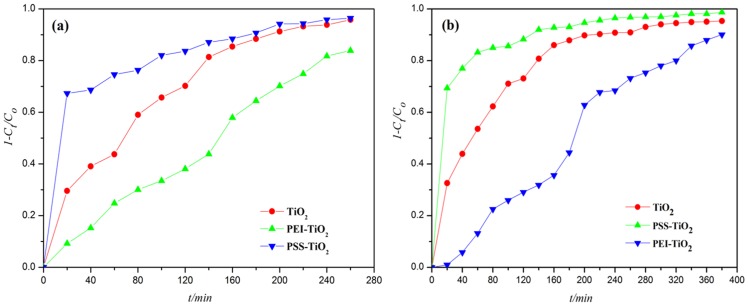
Photocatalytic degradation with time under UV light in the presence of TiO_2_, PEI-TiO_2_ and PSS-TiO_2_ photocatalysts of: (a) Methylene blue; (b) Rhodamine B.

Methylene blue (MB) is often used as a model contaminant of water for photocatalytic reaction test. And rhodamine B (RhB) is a typically industrial dye. The photodegradation was started after the thorough adsorption of dyes onto the photocatalyst in darkness. And the decolorization of dyes occurred with ultraviolet irradiation time. As shown in Fig. 7, the degradation efficiency on MB and RhB by photocatalysts, such as PEI-TiO_2_, PSS-TiO_2_ and TiO_2_, were much similar, that is, the degradation efficiency of PSS-TiO_2_ higher than that of TiO_2_. And the degradation efficiency of PEI-TiO_2_ is relatively lower than that of TiO_2_. Fig. 7 (a) showed that 0.96 degradation efficiency of PSS-TiO_2_ was observed on MB dye, a little higher than 0.95 and 0.84 of the pure TiO_2_ and PEI-TiO_2_, respectively, within 260 min of UV-light irradiation. Moreover, the PSS-TiO_2_ photocatalyst exhibited more efficient photo-degradation on MB in the initial stage. For example, 0.67 of MB was degradated by PSS-TiO_2_ within 20 min. In contrast, only 0.30 and 0.09 of MB were degraded by pure TiO_2_ and PEI-TiO_2_ photocatalysts within the same time. Fig. 7 (b) showed the similar results of degradation of RhB. The degradation efficiency of RhB was about 0.90, 0.95 and 0.99 with PEI-TiO_2_, TiO_2_ and PSS-TiO_2_, respectively, within 360 mins. This indicated that PSS-TiO_2_ nanoparticles showed relatively higher photocatalytic activity. The possible reason is that the negatively charged PSS-TiO_2_ easily adsorbed the positively charged MB by electrostatic attraction, as shown in Fig. 6(c), and made it effectively degradated. The relative lower degradation activity of MB over PEI-TiO_2_ than pure TiO_2_ nanoparticles is mainly ascribed to the electrostatic repulsion for MB shown in Fig. 6(b). For degradation of the RhB molecule, which contains both positively charged diethylamine groups and negatively charged carboxylate group, the carboxylate group is easily to adsorb onto Ti sites of TiO_2_ surface [45]. While, the positively charged diethylamine groups are prone to interact electrostatically with negative species, such as PSS anion occupied on TiO_2_ surface. Therefore, PSS-TiO_2_ exhibited notably accelerated degradation rate of RhB.

#### 3.3.3 Analysis of the photocatalytic performance of the nanoparticles

**Figure 8:**
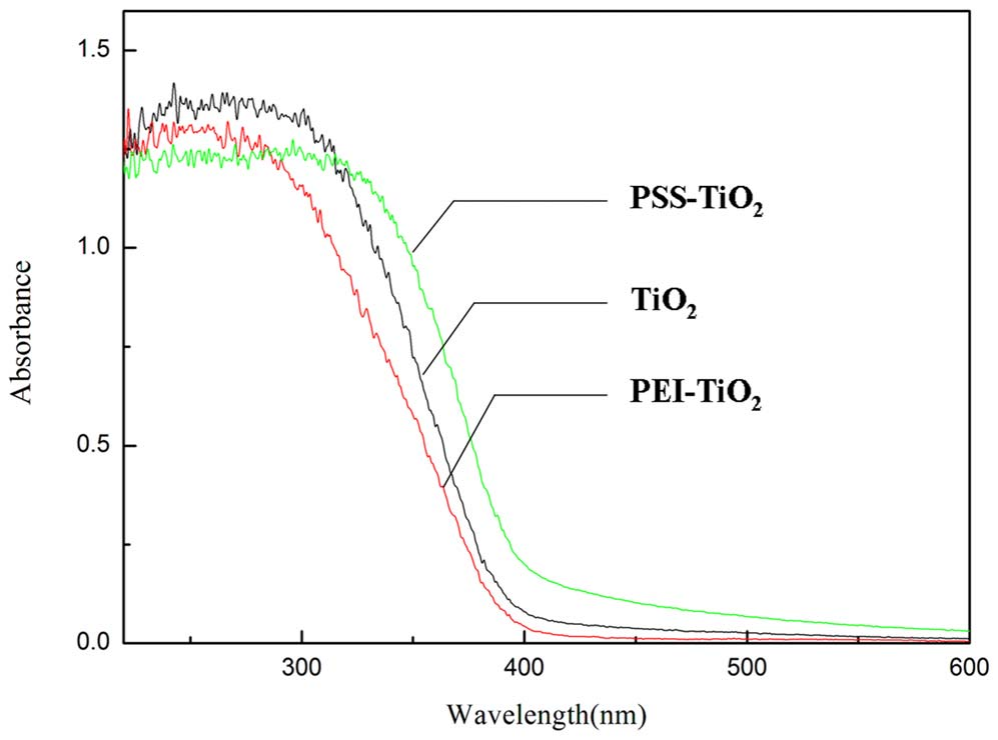
UV-vis diffuses reflectance spectra of TiO_2_, PEI-TiO_2_ and PSS-TiO_2_ photocatalysts.

Generally, the electrons of TiO_2_ would transfer from the valence band (VB) to conduction band (CB), when irradiated by photoelectrons with energy equal to/or larger than that of the band gap (*Eg*). Then, the photogenerated oxidative valence holes (h^+^) and reductive conduction electrons (e^−^) were produced [46]. In order to further expound the different photocatalytic performance of the above three samples, UV-vis diffuse reflectance spectra (DRS) were measured over the wavelength range from 200 nm to 600 nm. From the characteristic adsorption bands shown in Fig. 7, the band edge (*λ_g_*) and band gap energy (*E*_g_) of TiO_2_, PEI-TiO_2_ and PSS-TiO_2_ photocatalysts were shown in Table 2. Generally, TiO_2_ has a relatively high energy band gap (Eg≈3.2 eV) that can be excited under UV irradiation [47]. Here, the pure TiO_2_ sample showed the typical adsorption of anatase with an intense transition in the UV region of the spectrum, which was due to the promotion of the electron from the valence band to the conduction band in Fig. 8. The energy band gap was calculated about 2.88 eV as in Table 2. This *Eg* was further lowered to 2.44 eV by PSS modification of TiO_2_, in which the spectra band of the PSS-TiO_2_ nanoparticles exhibited red shift in the UV-vis range of 300–500 nm. This red shift indicated that the PSS with π-conjugated structure of aromatic ring has high electron mobility [48]. The high photodegrdation of dye molecules in Fig. 7 may be attributed to the efficient charge separation of the electrons (e^−^) and hole (h^+^) pairs at the interfaces of PSS and TiO_2_ which is related to the red shift. However, the spectra band of PEI modified TiO_2_ exhibited a blue shift with larger *E_g_* of 2.67 eV. This blue shift is possibly due to n-σ* transition of imine (-NH) in PEI macromolecules [49]. Consequently, the narrow band gap of PSS-TiO_2_ in Fig. 7 made it an enhanced photocatalytic efficiency of TiO_2_ under UV-light.

**Table 2:** Band edge (*λg*) and band gap energy (*E_g_*) of TiO_2_, PEI-TiO_2_ and PSS-TiO_2_ photocatalysts.

Sample	*λ_g_*/nm	*E*_g_/eV
TiO_2_	499.0	2.88
PEI-TiO_2_	464.5	2.67
PSS-TiO_2_	621.36	2.44

**Figure 9:**
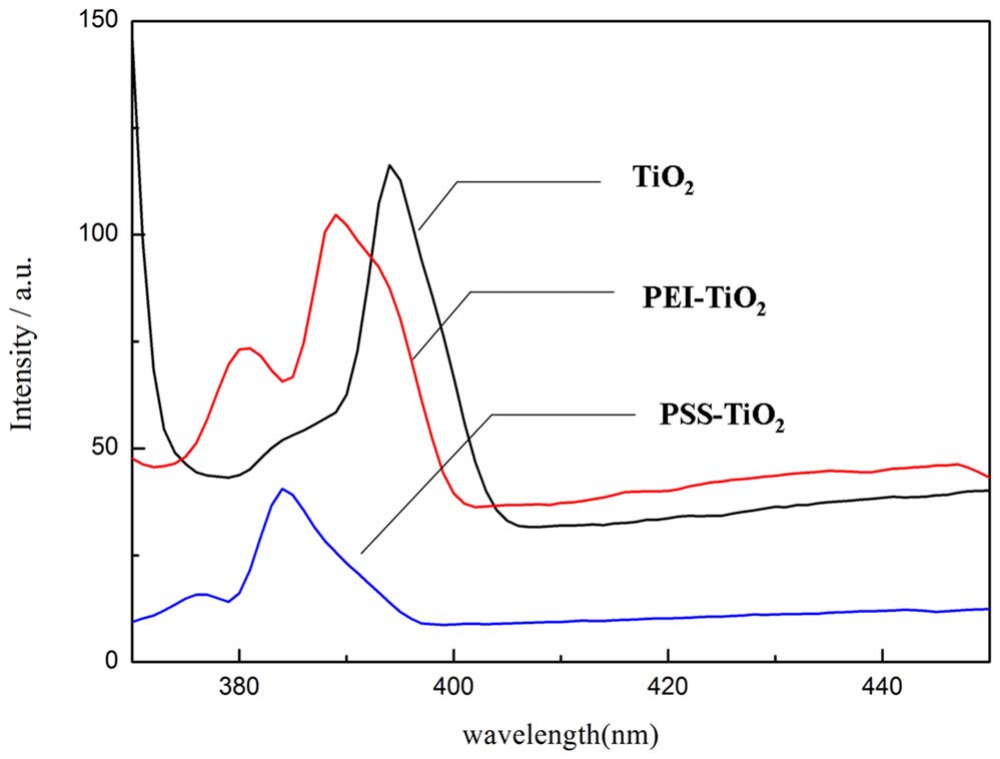
PL emission spectra (excited at 320 nm) of TiO_2_, PEI-TiO_2_ and PSS-TiO_2_ photocatalysts.

The photoluminscence (PL) emission, resulted from the recombination of excited electrons and holes further indicated the different photocatalytic efficiency as shown in Fig. 9. The broad-band emission spectra at around 375–410 nm are attributed to the luminescence signal, which indicated that the polyelectrolyte modification just affected the response range and intensity of the PL spectra, not causing new luminescence phenomenon in the PEI-TiO_2_ and PSS-TiO_2_ nanoparticles. In comparison with that of pure TiO_2_, the peak at about 396 nm for pure TiO_2_ was attributed to the excitation luminescence of free electrons from the CB bottom. After PSS modification, the PL intensity of TiO_2_ nanoparticles is significantly decreased, indicating that the PSS modification lowered recombination rate of electron/hole of TiO_2_ samples. However, the PEI-TiO_2_ nanoparticles displayed the higher PL intensity, indicating the higher recombination rate and *Eg* than PSS-TiO_2_. This difference was also probably due to the *E_g_* differences between π-π* transition of PSS and the n-σ* transition of imine (-NH) group of PEI polyelectrolyte, in accordance with that in Table 2.

**Figure 10:**
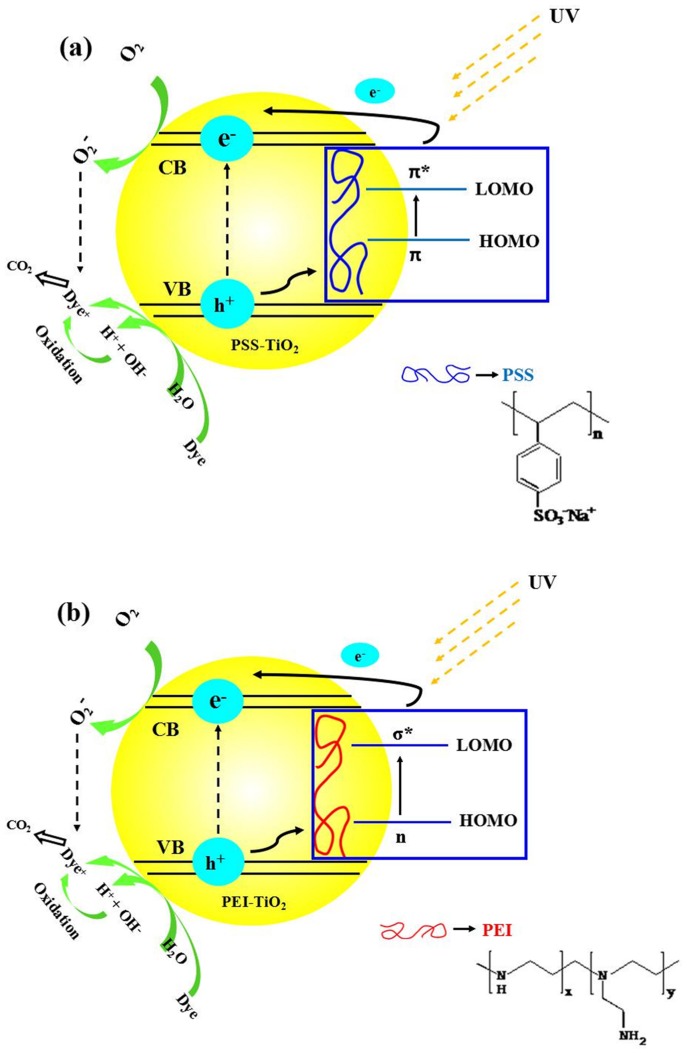
Schematic illustration of photocatalytic activity of (a) PSS-TiO_2_, (b) PEI-TiO_2_ nanoparticles for dye degradation under UV-light.

For better understanding of the photocatalytic activity differences between PEI-TiO_2_ and PSS-TiO_2_ photocatalysts, we used schemes of Fig. 10 to illustrate the dye degradation over the photocatalysts surfaces. Fig. 10 (a) indicated that PSS with aromatic groups showed higher electron mobility, while the VB position of TiO_2_ was lower than the highest occupied molecular orbital (HOMO) of PSS. Therefore, the hole (h^+^) was easily formed by e^−^ electron transference from VB of TiO_2_ to HOMO of PSS for e^−^ regeneration when TiO_2_ absorbed UV light. The generated e^−^ from PSS can be excitated from HOMO to the lowest unoccupied molecular orbital (LUMO) of PSS, and then the excitated e^−^ transferred to the CB of TiO_2_. Thus, more and more photogenerated e^−^ and h^+^ formed on TiO_2_ nanoparticles. The photogenerated e^−^ was so active that it can react with O_2_ to generate ·O_2_^−^. Meanwhile, the generated h^+^ can react with OH^–^ or H_2_O to generate ·OH. These radicals would react with dye molecules, and produce organic radicals or other intermediates. Finally, the radicals and intermediates were oxidized into CO_2_ and H_2_O. PSS of the TiO_2_ nanoparticles participated in charger transferring and favored enhancing the photocatalytic activities: e^−^ moved to the opposite direction from h^+^, reducing the recombination of photogenerated e^−^-h^+^ and making charge separation more efficient. The photogenerated e^−^-h^+^ pairs would favor the charge separation and the formation of oxyradicals (O_2_·, OH·). Compared to PSS-TiO_2_, the transition energy of n-σ* of PEI was much higher than that of π-π* of PSS, as shown in Fig. 10 (b). Hence, the negatively charged PSS-TiO_2_ exhibited better photocatalytic activity than pure TiO_2_ and positively charged PEI-TiO_2_.

## Conclusions

In conclusion, the anatase TiO_2_ nanoparticles were prepared by simple method of LTPPP, under a room temperature and atmospheric pressure. The modification of PEI and PSS has little effect on the anatase crystallite phase of TiO_2_. The zeta potential of nanoparticles can be effectively tuned by the variation of pH and the amount of polyelectrolytes during the synthesis process. Furthermore, PSS modified TiO_2_ nanoparticles exhibited the highest degradation efficiency of Methylene blue and Rhodamine B, in comparison with pure TiO_2_ and PEI-TiO_2_ nanoparticles. The higher photocatalytic activity of the PSS-TiO_2_ was attributed to the well adsorption of dye molecules, the reduced band gaps and lower recombination rate of electron/hole. This work would provide a simple and facile strategy to obtain various TiO2-based functional materials, for which is of significance to extending the practical applications.
